# The Sunday Disease

**Published:** 1856-04

**Authors:** 


### THE SUNDAY DISEASE.

A happy looking, honest-faced friend of ours, says benevolently, about sleeping in church: "When drowsiness is felt stealing over the senses, and weaving a web before the mind, so that the word of truth can make no impress upon the memory, the patient must lift his foot several inches above the floor, and hold it there in suspense, *without support to the limb.* Repeat the remedy as often as the attack comes on, by frequent use none of its virtue is lost, and no injury can come from it.

"The philosophy of this remedy it is needless to explain. I have tried it and found it effectual."

But what becomes of the sermon when one is trying to hold his foot up? We have a better remedy. Let your minister be a good man, let him be promptly, regularly, and handsomely paid, so that his whole soul, heart and mind, may be concentrated in the work of the ministry, and with forty minute discourses, and the whole services short of two hours, there will be no danger of drowsiness, under that man's ministrations. We try this remedy every Sunday in our Fifth Avenue church and find it NEVER FAILING.

